# Complete genome sequence of GII.9 norovirus

**DOI:** 10.1007/s00705-021-05257-x

**Published:** 2021-10-30

**Authors:** Zilong Zhang, Danlei Liu, Zilei Zhang, Peng Tian, Shenwei Li, Qingping Wu, Dapeng Wang, Zhengan Tian

**Affiliations:** 1 Shanghai International Travel Healthcare Center, Shanghai Customs District P. R. China, Shanghai, China; 2 State Key Laboratory of Applied Microbiology Southern China, Guangdong Provincial Key Laboratory of Microbial Culture Collection and Application, Guangdong Open Laboratory of Applied Microbiology, Guangdong Institute of Microbiology, Guangzhou, China; 3 Department of Food Science and Technology, School of Agriculture and Biology, Shanghai Jiao Tong University, Shanghai, China; 4 Inspection and quarantine technology communication department, Shanghai Customs College, Shanghai, 201204 China; 5 Produce Safety and Microbiology Research Unit, Western Regional Research Center, Agricultural Research Service-United States Department of Agriculture, Albany, CA USA

## Abstract

Norovirus is recognized as one of the leading causes of acute gastroenteritis outbreaks. Genotype GII.9 was first detected in Norfolk, VA, USA, in 1997. However, the complete genome sequence of this genotype has not yet been determined. In this study, a complete genome sequence of GII.9[P7] norovirus, SCD1878_GII.9[P7], from a patient was determined using high-throughput sequencing and rapid amplification of cDNA ends (RACE) technology. The complete genome sequence of SCD1878_GII.9[P7] is 7544 nucleotides (nt) in length with a 3’ poly(A) tail and contains three open reading frames. Sequence comparisons indicated that SCD1878_GII.9[P7] shares 92.1%-92.3% nucleotide sequence identity with GII.P7 (AB258331 and AB039777) and 96.7%-97.4% identity with GII.9 (AY038599 and DQ379715). The results suggested that SCD1878_GII.9[P7] is a member of P genotype GII.P7 and G genotype GII.9. This viral sequence fills a gap at the whole-genome level for the GII.9 genotype.

**Supplementary Information:**

The online version contains supplementary material available at 10.1007/s00705-021-05257-x.

## Introduction

Norovirus (NoV) is recognized as one of the leading causes of acute gastroenteritis outbreaks. NoV belongs to the family *Caliciviridae* and has a positive-sense ~7.5 kb RNA genome [1]. Phylogenetically, NoV can be segregated into 10 genogroups and further divided into genotypes based on amino acid sequence diversity in the VP1 gene. GII is the largest of the known genogroups, consisting of 26 genotypes, including 23 human NoV genotypes that are responsible for most epidemics, and three porcine NoV genotypes (GII.11/18/19) [2]. As the diversity of NoV increased through recombination, dual typing was proposed for NoV classification. Partial nucleotide sequences of the RNA-dependent RNA polymerase (RdRp) region of ORF1 are used for NoV P-type classification independently from genotype. A total of 37 P-types have now been identified for in GII viruses [2].

The first strain of genotype GII.9 virus (VA97207) was detected in Norfolk, VA, USA, in 1997 [3]. A partial genome sequence of this strain (a 3290-bp fragment including the complete ORF2 region) was uploaded to the GenBank database in 2001 (accession number AY038599) [3]. Compared with other genotypes, GII.9 strains have rarely been reported. Gelaw et. al. detected only one GII.9 strain in 450 clinical samples by RT-PCR and partially sequenced its VP1 gene (300 bp) [4]. The presence of GII.9 was also reported in wastewater in South Africa and oyster samples in Japan [5, 6]. Nevertheless, there was no submission of a GII.9 sequence to NoroNet from 2005 to 2016 [7].

## Materials and methods

In this study, a rare GII.9[P7] whole genome sequence was obtained from a clinical sample. An anal swab and epidemiological data were collected through the acute gastroenteritis (AGE) outbreak surveillance system monitored by Shanghai Customs. The patient was a 22-year-old Japanese female who traveled from India and arrived in Shanghai Pudong Airport on March 19, 2018. The patient had diarrhea and vomiting and was diagnosed as having AGE.

The majority of the whole viral sequence was determined using RNA-seq, and the ends of the viral genome were sequenced using a rapid amplification of cDNA ends (RACE) kit (Vazyme, Nanjing, China) (Supplementary Figs. S1 and S2) [8, 9]. The whole genomic sequence was then assembled and validated using CLC Genomics Workbench (https://digitalinsights.qiagen.com). The assembled viral genome sequence was genotyped using a web-based genotyping tool [10], and a phylogenetic tree was constructed using MEGA X [11]. The complete sequence, named SCD1878_GII.9[P7], was deposited in the GenBank database with the accession number MZ312111.

A total of 1976 human NoV genome sequences (6400-8500 bp) were obtained from ViPR on March 10, 2021 [12]. BioAider was used to remove sequences with sequence identity over 97% [13]. PhyloSuite was used to conduct, manage, and streamline the analyses [14]. Sequences were aligned using MAFFT [15]. The best partitioning scheme and evolutionary models for one pre-defined partition were selected using PartitionFinder2 [16], using the greedy algorithm and the AICc criterion. Maximum-likelihood phylogenetic trees were constructed using IQ-TREE [17] with the GTR+I+G4+F model and 20000 ultrafast bootstrap replicates, using the Shimodaira-Hasegawa-like approximate likelihood-ratio test [18].

## Results and discussion

The complete genome sequence of SCD1878_GII.9[P7] is 7544 nucleotides (nt) in length, with a 3’ poly(A) tail. As expected, the genome contains three open reading frames (ORFs) (Table 1). ORF1 can be cleaved into six nonstructural proteins: p48, NTPase, p22, VPg, Pro, and RdRp. The remaining two ORFs encode two structural proteins (VP1 and VP2). A comparison of the sequence against the reference sequence (NC_029646.1, GII.12[P12]) is summarized in Table 1.

**Table 1:** Comparison of the SCD1878_GII.9[P7] sequence with reference sequence NC_029646.1

	Begin	End	Coverage	Score	Concordance	Matches	Identity	I/D/M/F^*^	Stop Codons
NT	1	7518	100%	4816	32.50%	7479 (99.1%)	4987(66.1%)	27/39	
CDS									
ORF1	1	1700	100%	9172	78.30%	1692 (99.2%)	1261 (73.9%)	6/8/0/0	1
ORF2	1	536	100%	2712	71.10%	535 (99.3%)	351 (65.1%)	3/1/0/0	1
ORF3	1	260	100%	1086	66.70%	256 (98.5%)	159 (61.2%)	0/4/0/0	1
Proteins									
Nonstructural polyprotein (YP_009237897.1)	1	1700	100%	9172	78.30%	1692 (99.2%)	1261(73.9%)	6/8/0/0	1
p48 (YP_009238492.1)	1	330	100%	1541	65.90%	328 (97.6%)	209 (62.2%)	6/2/0/0	0
NTPase (YP_009238487.1)	1	366	100%	2126	87.60%	366 (100%)	299 (81.7%)	0/0/0/0	0
p22 (YP_009238488.1)	1	179	100%	536	46.10%	173 (96.6%)	78 (43.6%)	0/6/0/0	0
VPg (YP_009238489.1)	1	133	100%	832	92.10%	133 (100%)	120 (90.2%)	0/0/0/0	0
Pro (YP_009238490.1)	1	181	100%	1108	86.00%	181 (100%)	144 (79.6%)	0/0/0/0	0
RdRp (YP_009238491.1)	1	510	100%	3028	84.30%	510 (100%)	410 (80.4%)	0/0/0/0	0
VP1 (YP_009237898.1)	1	536	100%	2712	71.10%	535 (99.3%)	351 (65.1%)	3/1/0/0	1
VP2 (YP_009237899.1)	1	260	100%	1086	66.70%	256 (98.5%)	159 (61.2%)	0/4/0/0	1

*Insertions/deletions/misaligned/frameshifts

**Fig. 1:**
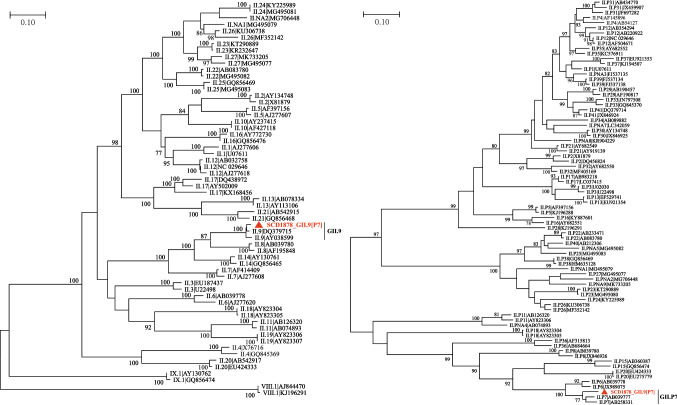
Phylogenetic tree of genotypes (left) and P-types (right) based on amino acid sequences of the complete VP1 protein and nucleotide sequences of the RNA-dependent RNA polymerase (RdRp) region respectively. The percentage of replicate trees (>75%) in the bootstrap test (500 replicates) is shown next to the branches.

Sequence comparisons indicated that SCD1878_GII.9[P7] shares 92.1%-92.3% and 96.7%-97.4% sequence identity with GII.P7 (AB258331 and AB039777) and GII.9 (AY038599 and DQ379715) at the nucleotide level in the RdRp gene and the amino acid level in the VP1 protein, respectively, suggesting that SCD1878_GII.9[P7] is a member of P genotype GII.P7 and G genotype GII.9 (Fig. 1). To investigate whether this isolate constitutes a new GII.P9 genotype, the RdRp region of DQ379715, AY038599 (GII.9), and reference sequences of GII.[P6]/[P7]/[P20]/[P15] were used to conduct evolutionary analysis by the maximum-likelihood method using the Kimura 2-parameter model. According to the "2-standard-deviation" (SD) criterion, where “the average distance between all sequences within a new genogroup or genotype and its nearest established cluster(s) should not overlap within 2 SD”, an overlap was observed between the average distance of this sequence and P6 or P7 sequences. Thus, the RdRp region of the related GII.9[P7] sequence could not form a new cluster in the phylogenetic tree, and the criterion of 2×SD could not be fulfilled [19, 20]. No significant difference was observed, and therefore, it could not be recognized as a new P type (Supplementary Fig. S3).

**Fig. 2:**
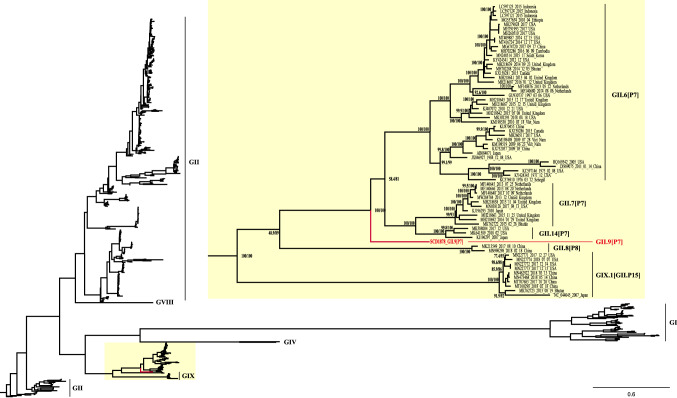
Maximum-likelihood phylogenetic tree for human NoV genome sequences (6400-8500 bp). The overall evolutionary relationship of SCD1878_GII.9[P7] to closely related NoV genogroups is shown in the tree on the left. An enlarged view of SCD1878_GII.9[P7]-related sequences is shown for the portion of the tree indicated by a yellow box. Ultrafast bootstrap values and Shimodaira-Hasegawa-like approximate-likelihood ratios are included in the node labels.

Phylogenetic analysis of whole genome sequences showed that SCD1878_GII.9[P7] clustered into a monophyletic clade with high confidence (bootstrap value = 100%, Fig. 2), together with three genotypes: GII.6[P7], GII.7[P7], and GII.14[P7]. Within the clade, SCD1878_GII.9[P7] formed its own distinct branch, confirming this sequence to be the first whole genome sequence of a GII.9[P7] genotype isolate. Potential recombination within the viral genome was screened using SimPlot, and no evidence for recombination events was detected in the genome of SCD1878_GII.9[P7] (Supplementary Fig. S4) [21].

The rapid development of sequencing technology has greatly facilitated virus monitoring. With the development of second- and third-generation sequencing technologies, discovering and analyzing longer viral genomes has become practical. Additional complete RdRp sequences or, ideally, complete genome sequences for all reference strains will help to improve the robustness of the present classification system [19]. Obtaining whole genome sequences of rare genotypes will not only enrich the database but also provide valuable information for analysis of evolution, as well as reference genome sequences for analysis of diversity, and screening for drug and vaccine development.

**Nucleotide sequence accession number** The GenBank accession number for norovirus SCD1878_GII.9[P7] is MZ312111.

## Supplementary Information

Below is the link to the electronic supplementary material.

Supplementary file1 (DOC 5032 kb)
