# Supplementary material for: Complete genome sequence of GII.9 norovirus
Source: Arch Virol. 2021 Oct 30;167(1):249–53. doi: 10.1007/s00705-021-05257-x (PMC8556859; doi:10.1007/s00705-021-05257-x)

**Figure.S1** Effective reads mapping of second-generation sequencing. Majority of whole viral sequence was generated through RNA-seq.


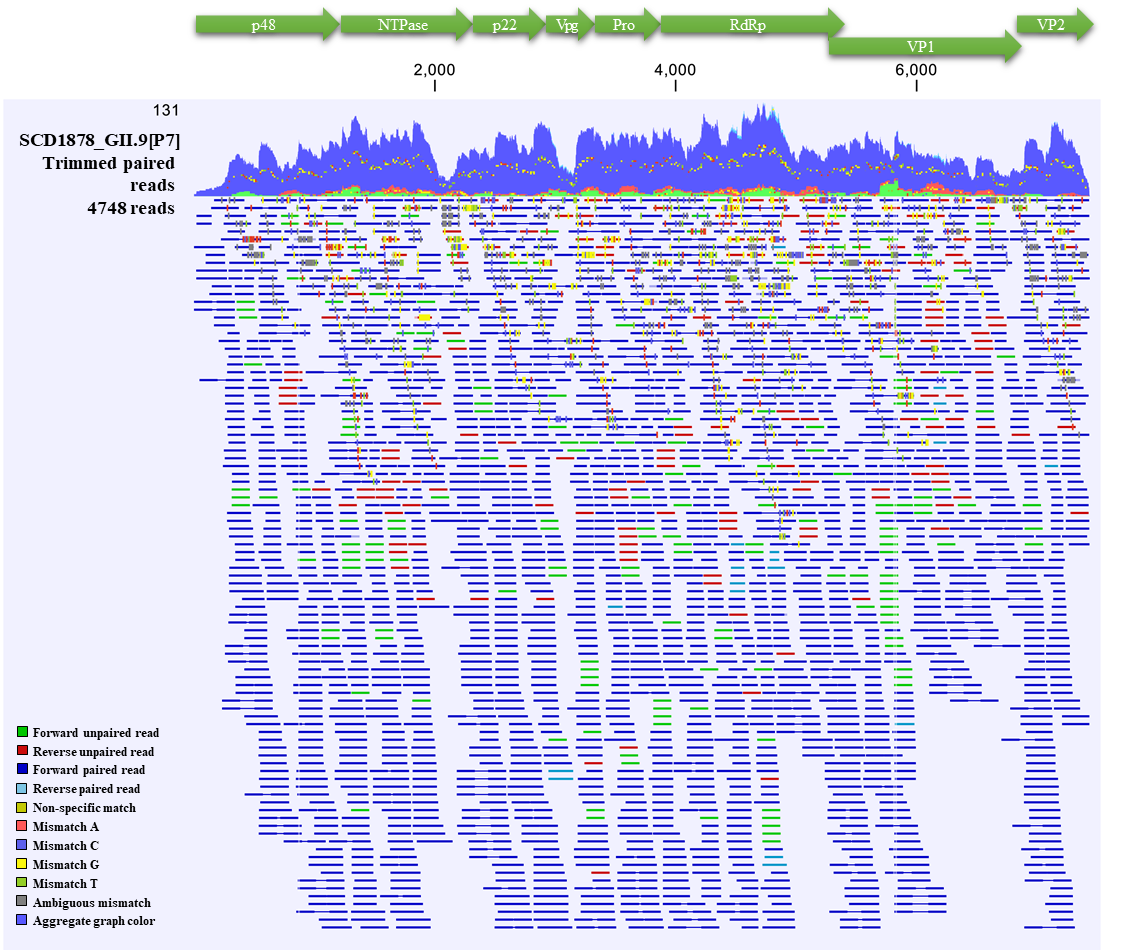


**Figure.S2** Sanger sequencing results of 5’ and 3’ rapid amplification of cDNA ends (RACE).


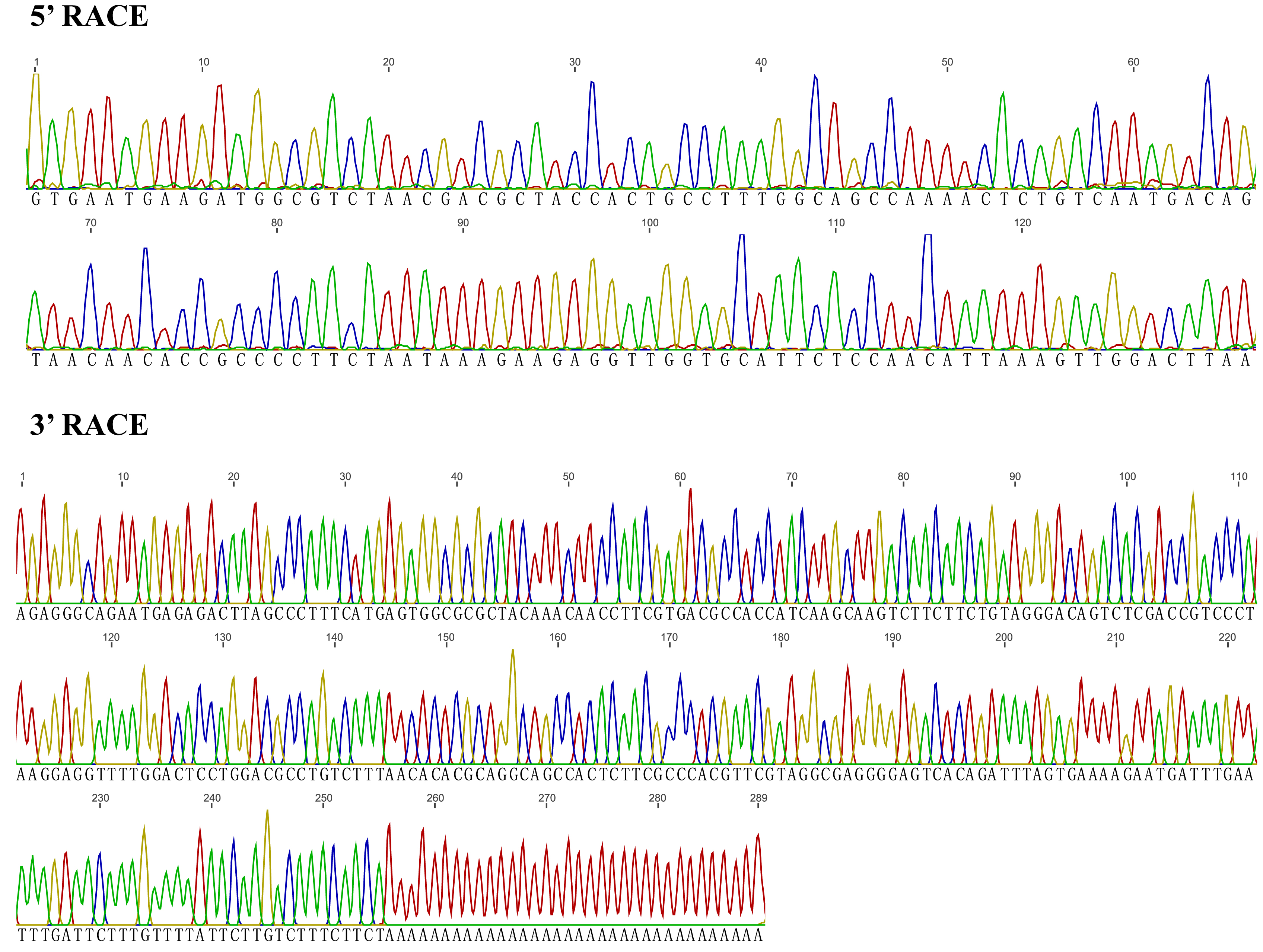


**Figure.S3** Evolutionary analysis of GII.P7 RdRp region by Maximum Likelihood method. RdRp regions of DQ379715, AY038599（GII.9）and reference sequences of [P6]\[P7]\[P20]\[P15] were included to conduct Evolutionary analysis. The evolutionary history was inferred by Maximum Likelihood method and Kimura 2-parameter model. In this study, 11 nucleotide sequences were involved. A total of 762 positions were included in the final dataset. Evolutionary analyses were conducted in MEGA X. RdRp sequence of GII.9[P7] could not be distinguished from P6 and P7 effectively.


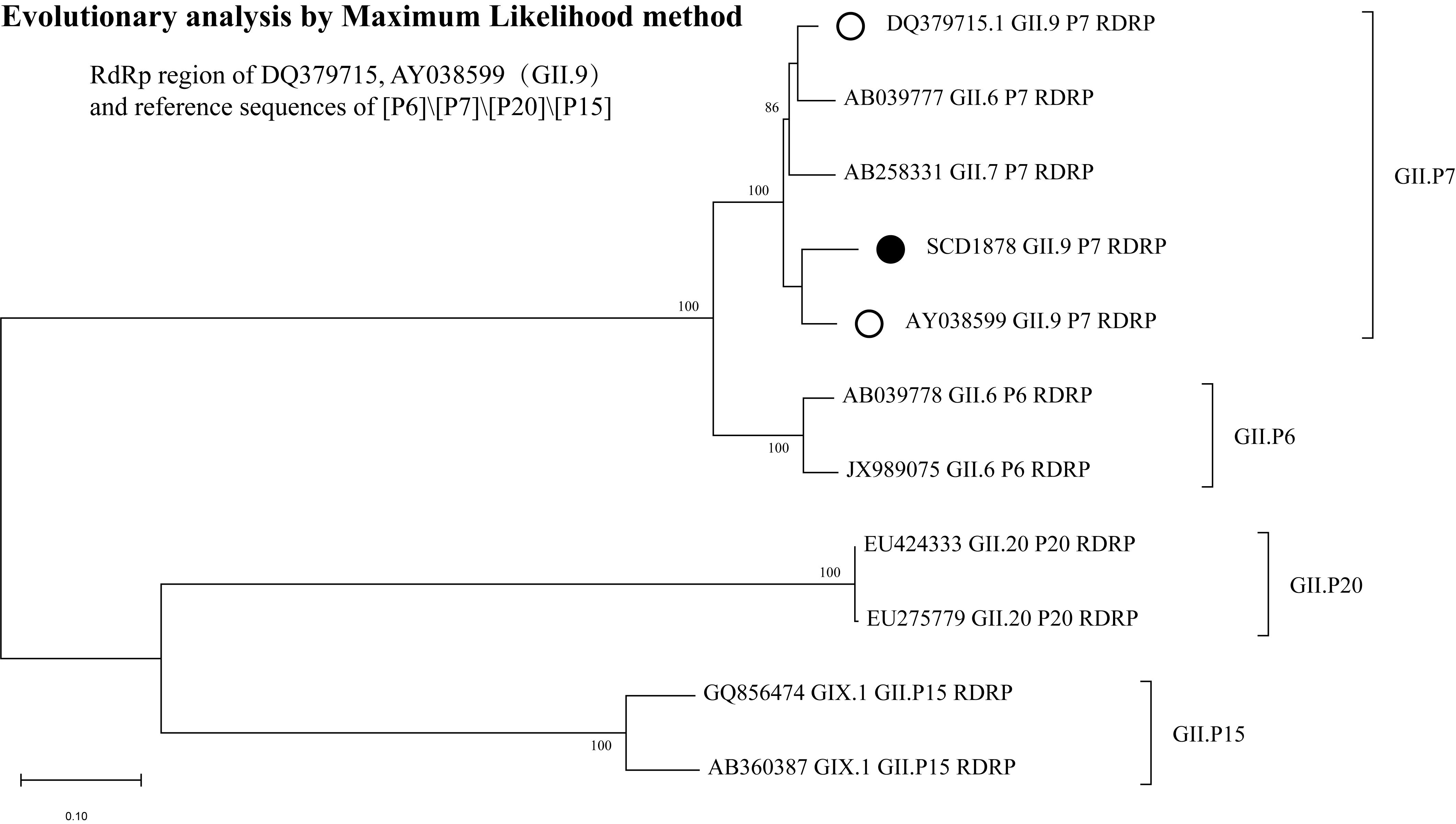


**Figure.S4** Similarity plot based on the genome sequence of SCD1878_GII.9[P7]. Genome sequences of GII.P6, GII.P7 and previously reported GII.9[P7] (DQ379715 and AY038599) were used as reference sequences. No evidence for recombination events was detected in the genome of SCD1878_GII.9[P7].


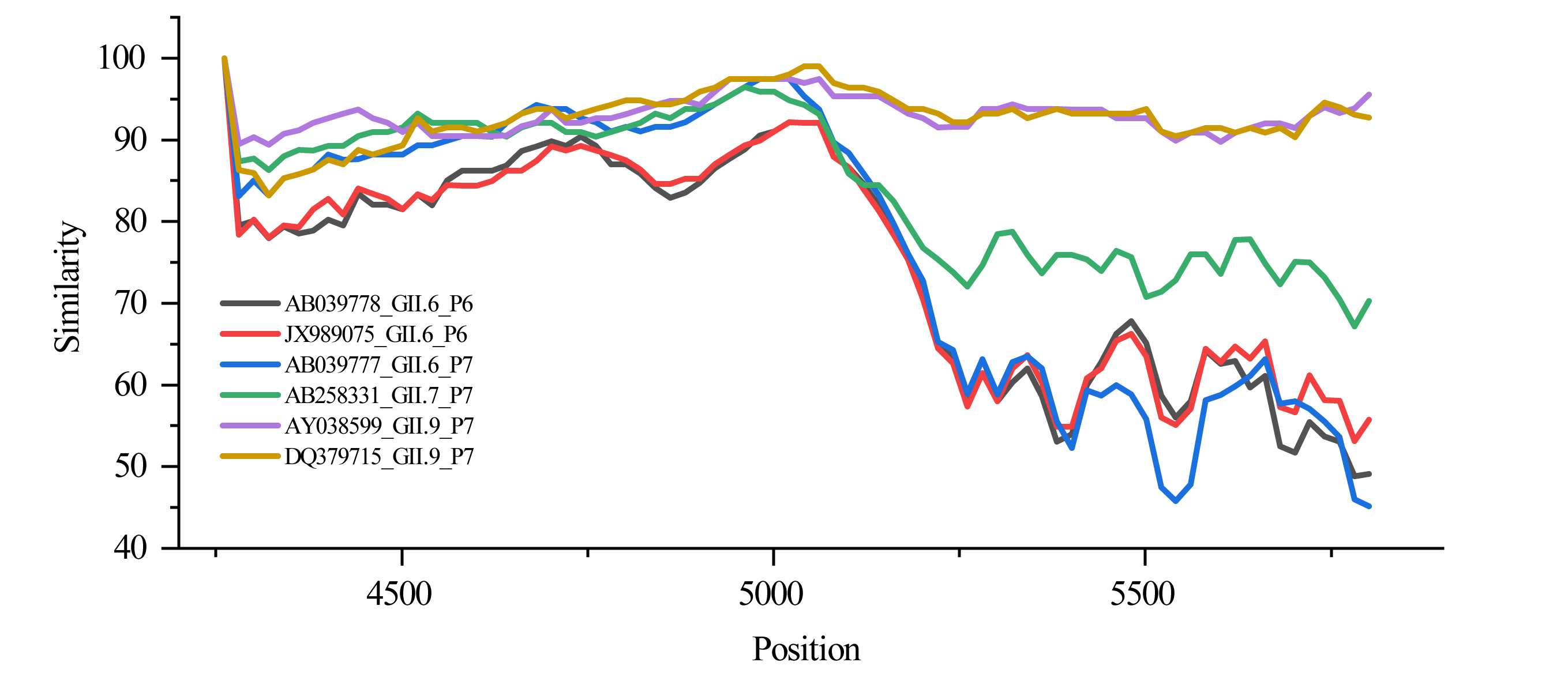

Supplement: Supplementary file 1 — Supplementary file1 (DOC 5032 kb) [file 705_2021_5257_MOESM1_ESM.doc]
